# Remarkable red colour vision in two Mediterranean beetle pollinators

**DOI:** 10.1242/jeb.250181

**Published:** 2025-06-30

**Authors:** Gregor Belušič, Sander B. de Hoop, Elena Bencúrová, Domen Lazar, Johannes Spaethe, Casper J. van der Kooi

**Affiliations:** ^1^ University of Ljubljana, Biotechnical Faculty, Department of Biology, 1000 Ljubljana, Slovenia; ^2^ University of Groningen, Groningen Institute for Evolutionary Biology, 9700 AB Groningen, The Netherlands; ^3^ University of Würzburg, Biocenter, Chair of Bioinformatics, 97074 Würzburg, Germany; ^4^ University of Würzburg, Biocenter, Chair of Behavioral Physiology and Sociobiology, 97074 Würzburg, Germany

**Keywords:** Glaphyridae, Flower colour, Visual ecology, Spectral sensitivity, Electrophysiology, Behavioural experiment, Scarabaeoidae, Poppy guild flowers

## Abstract

Beetles are one of the most species-rich insect orders and a primeval pollinator group, but much remains unknown about their sensory ecology. Glaphyrid beetles have a strong association with specific Mediterranean flowers, especially red, bowl-shaped flowers, suggesting an ability to see red colours. What is the physiological basis of the red sensitivity in Glaphyridae, and how does their ability to see red colours shape flower evolution in the Mediterranean? We investigated the glaphyrids *Pygopleurus chrysonotus* and *Pygopleurus syriacus* using electrophysiology, behavioural experiments and colour trapping. Intracellular recordings of photoreceptors revealed the presence of four photoreceptor types with peak sensitivities in the UV, blue, green and red wavelength ranges. Experiments in the field with *P. chrysonotus* demonstrated the behavioural use of colour vision to detect red targets as well as a clear preference for red colours. Glaphyridae represent an emerging model system for studies on beetle visual ecology and evolutionary tuning of (flower) signal production and detection by pollinators.

## Teaser

**Summary:** Two Mediterranean glaphyrid beetle pollinators see, select and prefer red flower colours.

## INTRODUCTION

The ability to see red colours is remarkably rare among insects. Whereas sensitivity in the (ultra)violet, blue and green wavelength ranges is evolutionarily common in insects, only a few insect species have red-sensitive photoreceptors (λ_max_>600 nm) (reviewed by van der Kooi et al., 2021). The only insect group where red sensitivity is abundant is diurnal butterflies, owing to rhodopsins with peak absorption in the green–yellow range (540–600 nm), spectrally filtered by perirhabdomal screening pigments (Arikawa et al., 2003; Bernard, 1979; Blake et al., 2019; Perry et al., 2016). In addition to that in butterflies, red sensitivity is currently known to occur in some dragonflies and three families of beetles, i.e. Carabidae, Buprestidae and Glaphyridae (reviewed by van der Kooi et al., 2021).

The single species of Glaphyridae for which the spectral sensitivity is known is *Pygopleurus israeliticus.* It was assumed to have an ultraviolet, green and red photoreceptor class with peak sensitivities at 352, 536 and 628 nm (Martínez-Harms et al., 2012). Both the absence of a blue photoreceptor and the clearly red-shifted sensitivity of the long-wavelength photoreceptor are intriguing. In beetles, the ancestral state seems to be a dichromatic retina with UV and green receptors, such as found in darkling, bark and dung beetles (Jackowska et al., 2007; Wu et al., 2020; Yilmaz et al., 2022; Morgante et al., 2025), owing to the evolutionary loss of blue opsin. Multiple beetle families have restored trichromacy by gaining the blue receptor class through a UV opsin gene duplication and spectral shift of the duplicated opsin to the blue part of the spectrum (Sharkey et al., 2017). In jewel beetles (Buprestidae), the retina comprises a complete set of UV, blue, green and red receptors (Meglič et al., 2020; Sharkey et al., 2023). Whether such an elaborated substrate for multispectral colour vision has evolved in other beetle groups and, if so, how it links to beetle visual ecology remains unknown.

Glaphyrids have an intricate relationship with flowers, because pollen is their main food source. Glaphyrid colour vision seems to be linked to the colours of the flowers that they visit. Red flowers are exceedingly rare across most of the European flora, though in the Mediterranean basin red flowers have evolved repeatedly in unrelated plant groups, e.g. in Mediterranean *Papaver*, *Anemone*, *Tulipa* and *Ranunculus*. These red-flowered taxa, which are commonly referred to as ‘poppy guild’ flowers, are presumed to be pollinated by *Pygopleurus* beetles (Dafni et al., 1990; Keasar et al., 2010; León-Osper and Narbona, 2022). Conversely, some other glaphyrid taxa have associations with other flower colours; for example, some species of *Eulasia* and *Glaphyrus* are mostly found on yellow or violet flowers (Sabatinelli et al., 2020). The mutualistic interaction between glaphyrids and flowers, and particularly the adoption of pollen as their main food source, is considered the main driver of radiation in Glaphyridae, which are considered ‘intermediate scarabs’ (Sabatinelli et al., 2020). Flower-visiting Glaphyridae thus constitute an insect group with diverse visual ecologies, linked to the visual signals of their floral food sources.

Here, we investigated the flower-visiting Mediterranean beetles *Pygopleurus chrysonotus* (Brullé 1832; synonym: *Pygopleurus diffusus* Petrovitz 1958; Bollino et al., 2019) and *Pygopleurus syriacus* (Linnaeus 1758). Performing intracellular recording of photoreceptors, behavioural experiments and colour trapping in natural habitats, we addressed the following three questions: (i) what are the spectral sensitivities of *P. chrysonotus* and *P. syriacus* photoreceptors; (ii) does *P. chrysonotus* use colour or achromatic vision to discriminate flowers from the background; and (iii) what are its colour preferences in natural habitats? We found that both species of *Pygopleurus* have UV, blue, green and extreme-red shifted photoreceptors, that flower discrimination occurs through colour and not achromatic vision, and that they prefer red stimuli over other colour stimuli in natural habitats.

## MATERIALS AND METHODS

### Study species and collection sites

Beetle specimens were caught on flowers around Mount Olympus, Greece, on the Albanian coast of Lake Skadar and near Lehavim in southern Israel during the spring seasons in 2023 and 2024. The behavioural experiments took place in the same area in Greece over 2 weeks in April–May 2024. Beetles that were used for the electrophysiological experiments were shipped to Ljubljana within 24 h of collection in the field, and typically arrived in the lab the next day. As the family Glaphyridae has undergone extensive revisions during recent decades, we used DNA barcoding to identify our experimental animals, following the classification by Sabatinelli et al. (2020). Species identification was confirmed using DNA barcoding for the COI primer pair ‘Jerry and Pat’ (Simon et al., 1994), which was also used by Sabatinelli et al. (2020) (Supplementary Materials and Methods, Fig. S1).

### Electrophysiology

Beetles were anaesthetized with ice, glued with a mixture of dental plastic and beeswax into pipette tips and mounted on a mini goniometric stage, which also carried a 50 µm diameter Ag/AgCl wire that was inserted into the head capsule as a reference electrode. A small hole was cut into the ventral cornea with a razorblade chip for the recording microelectrode and covered with silicon vacuum grease. The stage with the insect was mounted into a large recording goniometer, which also carried the micromanipulator (Sensapex, Finland). Microelectrodes, made from 1.00/0.50 mm outer/inner diameter borosilicate glass pipettes, with resistance 100–150 MΩ, were made on a P-2000 laser puller (Sutter Instrument, Novato, CA, USA) and loaded with 3 mol l^−1^ KCl. Intracellular recordings were obtained with a SEC-10LX amplifier (NPI, Tamm, Germany), operating in bridge mode and in current clamp mode at 20 kHz switching frequency. Flash stimulation was provided by a LED array (Belušič et al., 2016) and a 75 W xenon arc lamp (Cairn Research, Faversham, UK), filtered with a monochromator (B&M Optik, Limburg an der Lahn, Germany), both adjusted to emit an equal number of photons per flash (isoquantal intensity ∼2×10^14^ photons cm^−2^ s^−1^, maximal intensity at 500 nm ∼10^15^ photons cm^−2^ s^−1^). The two light sources were projected coaxially on the eye. The goniometric stage was carefully rotated to bring the impaled photoreceptor into the optical axis of the stimulator and the aperture of the stimulating beam was closed to ∼1.5 deg, i.e. restricted to within the spatial field of the targeted photoreceptor. The recording platform is presented in Fig. S2. The signals were digitized with a Micro1401 mk II interface (Cambridge Electronic Design, Cambridge, UK), recorded and analysed with WinWCP 5.5.4 (see also Meglič et al., 2020). Recordings were performed in *n*=3 *P. chrysonotus* (3 males) and *n*=2 *P. syriacus* (1 female, 1 male) specimens.

### Behavioural experiments and colour trapping in natural populations

The presence of certain photoreceptor types is not direct evidence of colour vision. It is possible that beetles use achromatic (brightness) cues to find (red) flowers. ‘True colour vision’ is a psychophysical phenomenon that can only be demonstrated by means of behavioural experiments that test whether a viewer can discriminate between colours of a specific spectral composition irrespective of the stimuli's relative intensity. We performed just such an experiment, inspired by the seminal work of Von Frisch (1914) and others (Arikawa et al., 2021; Kelber et al., 2002). We tested for colour vision in *P. chrysonotus* by releasing individuals in a small net tent with an array of artificial stimuli. The array consisted of three red stimuli and 12 grey stimuli of different intensities (three of each intensity). The stimuli, round coloured paper of 6 cm diameter, were presented on sticks at about 20 cm above the grassy ground, which was covered with uniform grey paper (#122, Canson, Annonay, France). The experiments took place under the full sun in the afternoon, when beetles are most active in the field. The first visit to an artificial stimulus was recorded. Eleven beetles were tested with red versus light greys, and 12 beetles were tested with red versus dark greys. The red stimulus was made from tinted drawing paper (ruby red #22, Ludwig Bär, Kassel, Germany), the grey stimuli were made by printing different shades (0%, 10%, 20%, 30%, 40%, 50%, 60% and 70%; light grey #80, Ludwig Bär) on paper with a laser printer (MS331, Lexmark, Lexington, KY, USA). Reflectance spectra are provided in Fig. S3. Stimuli were randomly reordered after every visit, and the stimulus that was touched by the beetles was replaced to avoid any olfactory effect.

To test beetle colour preferences in natural conditions, we performed colour trapping experiments (Besana et al., 2025; Cavaletto et al., 2021; Streinzer et al., 2019). Over a period of 4 days with ambient temperatures of about 25°C, we put colour traps out in natural populations. Colour traps consisted of five colours: white, purple, blue, yellow and red (Fig. S3), with three sets of each colour spread out in a diverse meadow of flowering plants. The traps were made of cylindrical transparent Plexiglas cups (7 cm height, 7 cm diameter), placed on top of a 20 cm metal stick, filled with water mixed with an odourless detergent and the coloured stimulus (cross insertions made of laminated colour paper) in the centre. Trapping was carried out for 4–7 h per day, always including midday when glaphyrid beetles are most active. The total period of trapping was 23.5 h. All caught insects were kept in ethanol and identified in the lab in Würzburg.

## RESULTS

### Four photoreceptor types and synaptic inhibition in the red photoreceptor

**Fig. 1.**
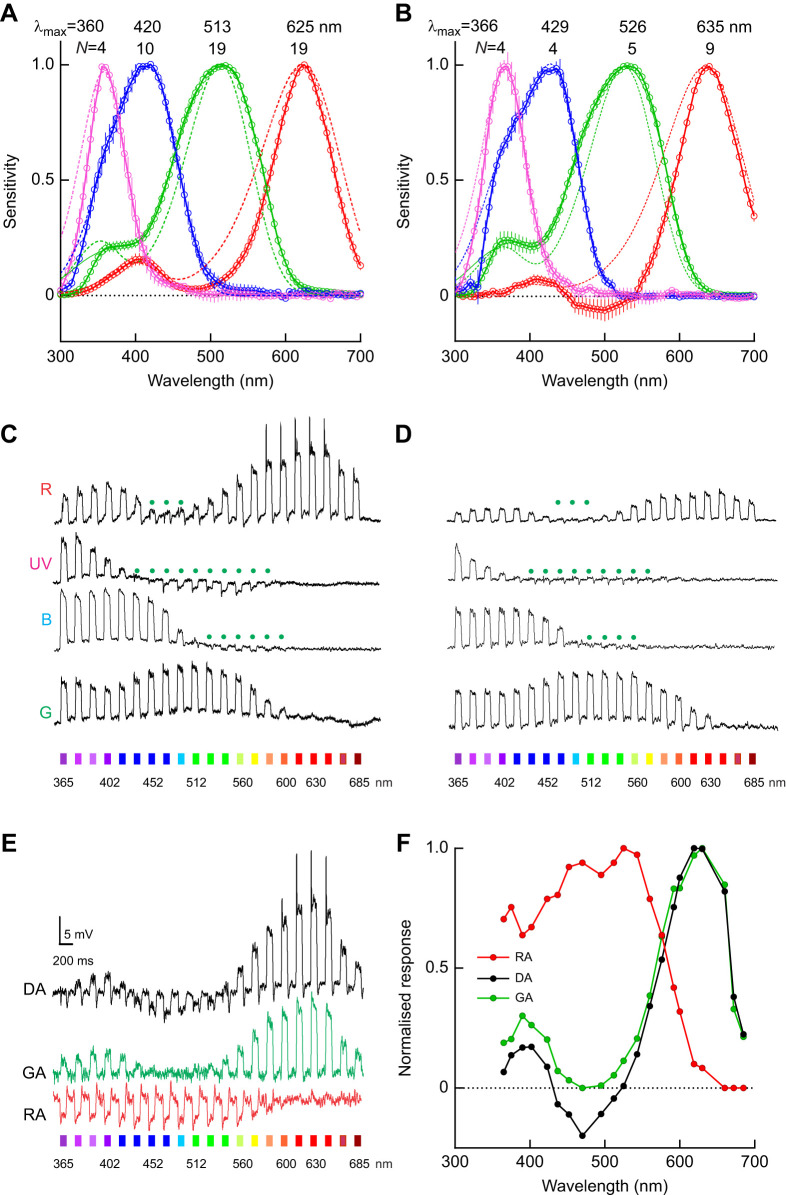
**Electrophysiological analysis of glaphyrid beetle photoreceptors.** (A,B) Normalised spectral sensitivity of four classes of photoreceptors in *Pygopleurus chrysonotus* and *Pygopleurus syriacus*. Bold curves show means±s.e.m., with number of recorded cells *N*; dashed curves are rhodopsin templates with peak wavelength λ_max_. (C,D) Voltage traces from the red- (R), UV-, blue- (B) and green- (G) sensitive photoreceptors in (C) *P. chrysonotus* and (D) *P. syriacus*, stimulated with a spectral series of flashes with equal quanta at the indicated wavelengths. Opponent responses are indicated with green dots. (E) Voltage traces from a red-sensitive photoreceptor in *P. syriacus*, stimulated with a spectral series of flashes in the dark-adapted (DA), green-adapted (GA) and red-adapted (RA) state. (F) Normalised response amplitudes from E.

Our recordings in the retina of *Pygopleurus* revealed highly similar sets of four classes of spectral photoreceptors in the two species studied. In addition to the three known spectral classes (UV, green, red), found previously in *P. israeliticus* (Martínez-Harms et al., 2012), both *P. chrysonotus* and *P. syriacus* have blue-sensitive photoreceptors, peaking at ∼430 nm (Fig. 1). The blue photoreceptor type is presumably derived from the UV receptors by gene duplication and spectral shift (Sharkey et al., 2017). The UV-, green-, blue- and red-sensitive photoreceptors together form a complete spectral set for potential tetrachromatic vision. The most frequently impaled receptors were of the green receptor class (total number of impaled green-sensitive cells with the quickly scanned spectral sensitivity using the LED array: ∼80), suggesting that they are the main input into the achromatic part of the visual pathway, as found in other insects (Wakakuwa et al., 2005; Skorupski and Chittka, 2010; van der Kooi and Kelber, 2022).

The shapes of the spectral sensitivity curves were fitted with the Stavenga–Smits–Hoenders rhodopsin template (Stavenga et al., 1993). The spectral sensitivity numerical data for the two species are provided in Dataset 1. The fitting procedure omitted values below 350 nm, because all measured sensitivities were lower than the modelled values, presumably as a result of UV filtering by the beetle cornea (Ilić et al., 2016). The green receptors' sensitivity spectrum was broader than the template, indicating self-screening, i.e. increased absorption of light in long rhabdomeres at wavelengths off peak sensitivity (Smakman and Stavenga, 1986). The sensitivity spectrum of the red receptors was distinctly narrower than the template (half-width: sensitivity curve ∼90 nm versus template ∼130 nm), indicating inter-photoreceptor opponency, specifically because the voltage traces of red receptor responses in the green part of the spectrum often showed light responses with negative polarity (Fig. 1C,D). Selective adaptation with monochromatic red light (630 nm) saturated the impaled red unit and isolated the opponent responses of green-sensitive photoreceptors, whereas adaptation with monochromatic green light suppressed the opponent responses and revealed the sensitivity of the red unit (Fig. 1E,F). Spectral-opponent signals from green photoreceptors could also be detected in UV- and blue-sensitive receptors (Fig. 1C,D).

### Colour vision and a preference for red colours in *P. chrysonotus*

**Fig. 2.**
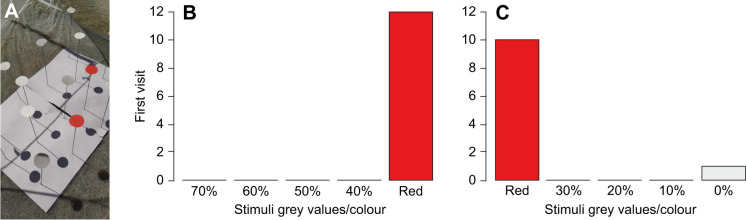
**Colour vision in *P. chrysonotus*.** (A) The test array with red and light grey stimuli. (B,C) Behavioural responses during tests with red and dark grey stimuli (B) and with red and light grey stimuli (C). Percentages indicate the degree of black printer ink coverage of the stimulus.

**Fig. 3.**
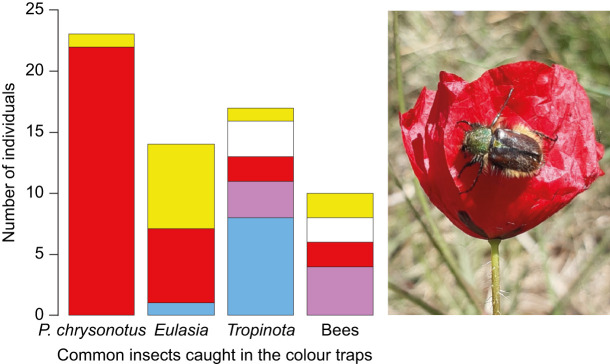
**Colour preference for insects caught in colour traps.** The colour of the bars represents the colour of the traps in which they were caught. The picture shows a *P. chrysonotus* beetle on a poppy flower in Greece.

Our behavioural experiments with 23 individuals clearly showed that *P. chrysonotus* uses colour vision and not achromatic vision to find red targets (Fig. 2). Ten out of 11 tested individuals chose red over light grey targets (χ^2^=34.9, d.f.=4, *P*<0.001) and 12 out of 12 tested individuals chose red over dark grey (χ^2^=48, d.f.=4, *P*<0.001). Colour trapping in natural populations revealed that *P. chrysonotus* beetles prefer red over white, blue, violet and yellow (Fig. 3). In total, we caught 64 individual insects, 54 of which were beetles. Twenty-two out of 23 caught *P. chrysonotus* individuals were caught in red traps (χ^2^=82.4, d.f.=4, *P*<0.001). Colour preferences are less pronounced in other common, co-occurring flower-visiting beetles, such as *Tropinota* sp. (Scarabaeidae) and *Eulasia* sp. (Glaphyridae), as well as various bee species (Fig. 3).

## DISCUSSION

We investigated colour vision in *P. chrysonotus* and *P. syriacus*, two ecologically important beetles in the eastern Mediterranean, via retinal electrophysiology and behavioural experiments in their natural habitat. The two species have almost identical sets of spectral photoreceptors. Electrophysiology revealed four types of photoreceptors (UV, blue, green and red; putative tetrachromacy). Red photoreceptors enable the beetles to distinguish the colour contrast of red ‘poppy guild’ flowers from the background foliage and substrate. Perceived colour contrast is a key feature for effective discrimination of flowers (Kelber and Osorio, 2010; van der Kooi and Spaethe, 2022, 2025). The presence of spectral opponency in three out of four classes of beetle photoreceptors is highly similar to that of butterfly photoreceptors (Chen et al., 2020; Matsushita et al., 2022), indicating that the early visual pathway of Glaphyridae encompasses signal processing for colour vision. The magnitude of opponency somewhat varied among individuals and was stronger in *P. syriacus* than in *P. chrysonotus*, which resulted in slightly shifted spectral sensitivity peaks, albeit this would benefit from further study. Behavioural tests revealed that *P. chrysonotus* uses colour vision in the field, although whether it uses all four photoreceptor classes similarly in natural settings, i.e. functionally tetrachromacy, requires further study.

Our discovery of a blue photoreceptor in *P. chrysonotus* and *P. syriacus* is in line with results of buprestid beetles (Lord et al., 2016; Meglič et al., 2020; Sharkey et al., 2023) but contradicts previous results found for the congener *P. israeliticus.* Martínez-Harms et al. (2012) recorded only clear responses for UV, green and red photoreceptors for *P. israeliticus*. Our samples of *P. syriacus* were collected from the same source region and habitat from which Martínez-Harms et al. (2012) collected their beetles. Given that the Glaphyridae phylogeny is only beginning to be understood (Sabatinelli et al., 2020), we should treat former species identifications with caution. *Pygopleurus* beetles are difficult to identify based on morphology alone and only males can reliably be identified (Bollino et al., 2019; Sabatinelli et al., 2020), which is why we identified our beetles using genetic markers (Fig. S1). The discrepancy in the number of photoreceptor classes may be explained by the stochastic nature of single cell recordings. Indeed, Martínez-Harms et al. (2012) acknowledged that they could not rule out the possible presence of a blue photoreceptor type. We now confirm that a blue photoreceptor is present in at least two *Pygopleurus* species.

To the best of our knowledge, we are the first to experimentally validate the behavioural use of colour vision in a beetle (as per Von Frisch, 1914). The presence of several types of photoreceptors is commonly taken for granted as indicative that a species exhibits colour vision, though evidence that colours are distinguished from grey stimuli irrespective of intensity is needed to validate behavioural use of colour vision. Such conclusive evidence is available for comparatively few insect species: honeybees (Von Frisch, 1914), stingless bees (Spaethe et al., 2014), hawkmoths (Kelber and Henique, 1999; Kelber et al., 2002; White et al., 1994), butterflies (e.g. Swihart, 1971; Blackiston et al., 2011; Kelber and Pfaff, 1999; Kinoshita et al., 1999; Arikawa et al., 2021; Finkbeiner and Briscoe, 2021), three species of fly (reviewed by Lunau, 2014) and one beetle species (this study). Previous behavioural and/or electrophysiological evidence suggested the presence of colour vision and opponency in some beetle species (e.g. Booth et al., 2004; Doering et al., 2012), akin to what we have found for *P. chrysonotus*. Although *P. chrysonotus* uses colour vision and not achromatic vision for short-range visual tasks, it remains possible that more long-range detection relies primarily on achromatic cues, as seems to be the case for many insects (Giurfa et al., 1996; Meena et al., 2021; van der Kooi and Kelber, 2022).

The fact that *P. chrysonotus* can be used in field-based experiments and responds to artificial stimuli opens avenues for more detailed behavioural experiments on their visual ecology. This is particularly exciting given the ecological importance of beetles, which are immensely understudied given their species richness. Glaphyridae are particularly important for pollination of Mediterranean flowers (Bernhardt, 2000; Dafni et al., 1990; Keasar et al., 2010; Sabatinelli et al., 2020), and thus constitute a tractable model system for studies on the tuning of signal production (flower colour) and detection.

Glaphyrid beetles crucially depend upon pollen, and they commonly use flowers as sleeping and mating sites (Keasar et al., 2010). It is interesting that *P. chrysonotus* prefers and is specialised on red flowers (Fig. 3), because it should also be able to see more common floral colours such as yellow and white, assuming it uses all four photoreceptor types while foraging. A preference for red stimuli might have evolved because red sensitivity has enabled *Pygopleurus* species to exploit an ecological niche that is invisible to other flower-visiting insects. In other words, the ability to see red creates a private niche in colour space (*sensu* Lunau et al., 2011). Red, ‘poppy guild’ flowers may have specialised on pollination by red-sensitive glaphyrids if it is beneficial to reduce visibility to other insects (including pollen robbers) (e.g. León-Osper and Narbona, 2022) and/or if the pollination efficacy of glaphyrids is high. Experimental tests on the pollination efficacy of glaphyrids and on whether their colour preferences are innate or learned will help address this interesting case of co-evolution of flower colour and pollinator vision.

The apparent variation in visual ecology among closely related beetle taxa with similar ecological demands raises a tantalising evolutionary scenario. Flower colouration is generally assumed to adapt to the visual systems of pollinators and not vice versa (Chittka, 1996; van der Kooi and Ollerton, 2020), though it is tempting to speculate that this evolutionary scenario is not so unidirectional in Glaphyridae and their flowers. There is considerable variation in putative flower colour preferences within the three extant flower-visiting genera of Glaphyridae. In *Eulasia*, *Glaphyrus* and *Pygopleurus*, colour preferences vary among red, violet, white and yellow – sometimes even among sister species (Sabatinelli et al., 2020). The high degree of variation in colour preferences among closely related glaphyrids suggests that the physiological and/or behavioural basis for (red) colour vision is comparatively labile. This is corroborated by evidence on opsin evolution in beetles (Sharkey et al., 2017, 2021, 2023). The great diversity of flower colours in the Mediterranean, which is a biodiversity hotspot, together with the considerable variation in glaphyrid visual ecology makes it plausible that this pollinator's visual system adapts to flower colours more than commonly assumed.

## Supplementary Material

10.1242/jexbio.250181_sup1 Supplementary information

Dataset 1. Spectral sensitivity numerical data for the two species.
