# Supplementary material for: Remarkable red colour vision in two Mediterranean beetle pollinators
Source: J Exp Biol. 2025 Jun 30;228(12):jeb250181. doi: 10.1242/jeb.250181 (PMC12268171; doi:10.1242/jeb.250181)
Supplement: Supplementary information [file jexbio-228-250181-s1.pdf]

Supplementary Materials and Methods

The phylogenetic tree was created using the Maximum Likelihood (ML) method with the General Time Reversible (GTR) model of nucleotide substitutions [1]. The analysis comprised 13 nucleotide sequences with 857 positions in the final dataset. Nucleotide alignment was performed using Muscle alignment (method: UPGMA). Evolutionary rate differences among sites were modelled using a discrete Gamma distribution with 4 categories (+G, parameter = 0.1598). The tree with the highest log-likelihood score (-3.604,20) is shown, and bootstrap values, representing the percentage of replicate trees (1000 replications) in which the associated taxa clustered together, are indicated next to the branches [2].

For the initial tree in the heuristic search, the tree with the superior log-likelihood was selected between a Neighbour-Joining (NJ) tree [3] and a Maximum Parsimony (MP) tree. The NJ tree was generated based on pairwise distances computed using the GTR model. The MP tree had the shortest tree length among 10 MP tree searches, each performed with a randomly generated starting tree. The three was generated using the MEGA12 software [4], utilizing up to 3 parallel computing threads. For tree construction, the Nearest Neighbour Interchange (NNI) heuristic search method was employed with 1000 bootstrap replications for branch support estimation. Bootstrap values exceeding 70% are generally indicative of robust support for the clade, with a value of 100 representing the highest level of statistical confidence in the inferred evolutionary relationships. The sequences for the species are submitted in the GenBank under the accession numbers PV670937(*P. chrysonothus*) and PV682797 (*P.* cf. *syriacus*). The published sequence of *P. syriacus* slightly differs from ours, indicating a potential subspecies.

References

[1] Nei, M., & Kumar, S. (2000). *Molecular evolution and phylogenetics*. Oxford university press.

[2] Felsenstein, J. (1985). Confidence limits on phylogenies: an approach using the bootstrap. *evolution*, *39*(4), 783-791.

[3] Saitou, N., & Nei, M. (1987). The neighbor-joining method: a new method for reconstructing phylogenetic trees. *Molecular biology and evolution*, *4*(4), 406-425.

[4] Kumar, S., Stecher, G., Suleski, M., Sanderford, M., Sharma, S., & Tamura, K. (2024). MEGA12: Molecular Evolutionary Genetic Analysis version 12 for adaptive and green computing. *Molecular Biology and Evolution*, *41*(12), msae263.

Journal of Experimental Biology • Supplementary information

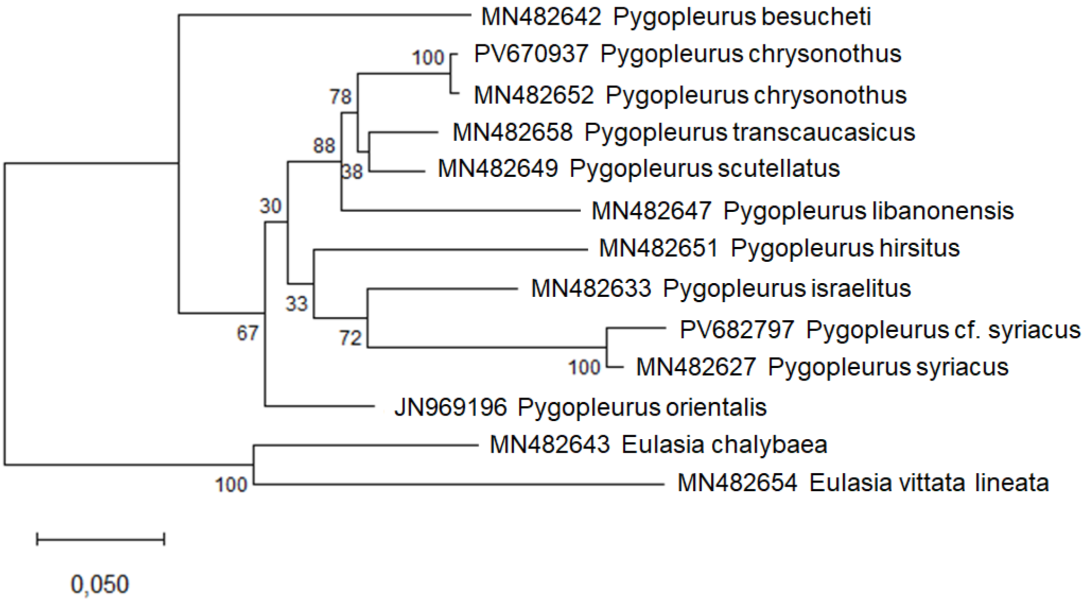

Fig. S1. Phylogenetic relatedness. GenBank accession numbers of sequences are given before the species names. The sequences for the species we submitted to GenBank are PV670937(*P. chrysonothus*) and PV682797 (*P.* cf. *syriacus*). Bootstrap values are shown on branches (1000 replicates).

Journal of Experimental Biology • Supplementary information

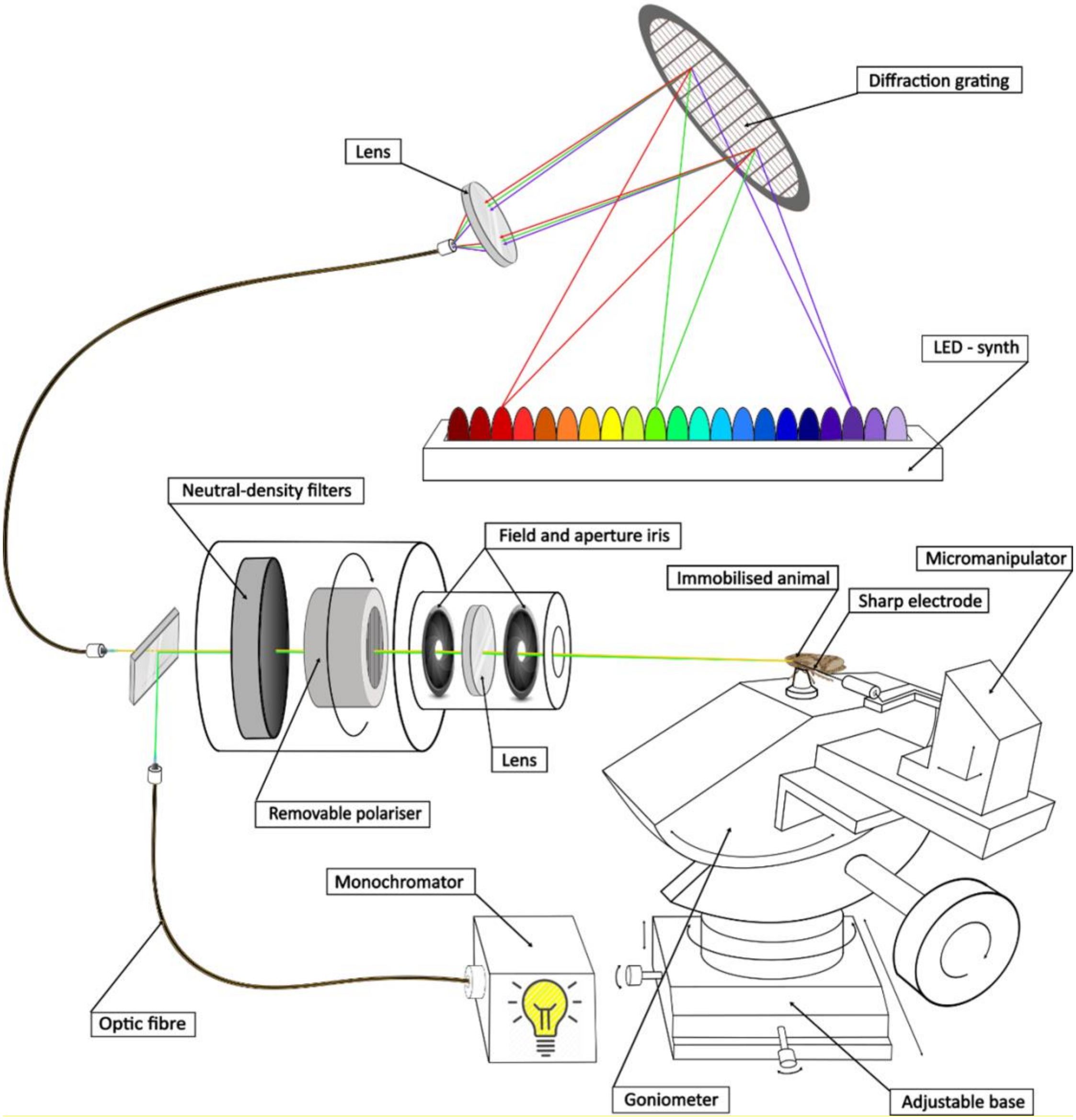

Fig. S2. Recording and stimulation set-up for single cell recordings in the retina. The goniometer with the animal and micromanipulator is at the bottom to the right. Flashes with defined wavelength, intensity and polarisation are created by the monochromator and LED array and projected coaxially to the eye in the centre of rotation of the goniometer.

Journal of Experimental Biology • Supplementary information

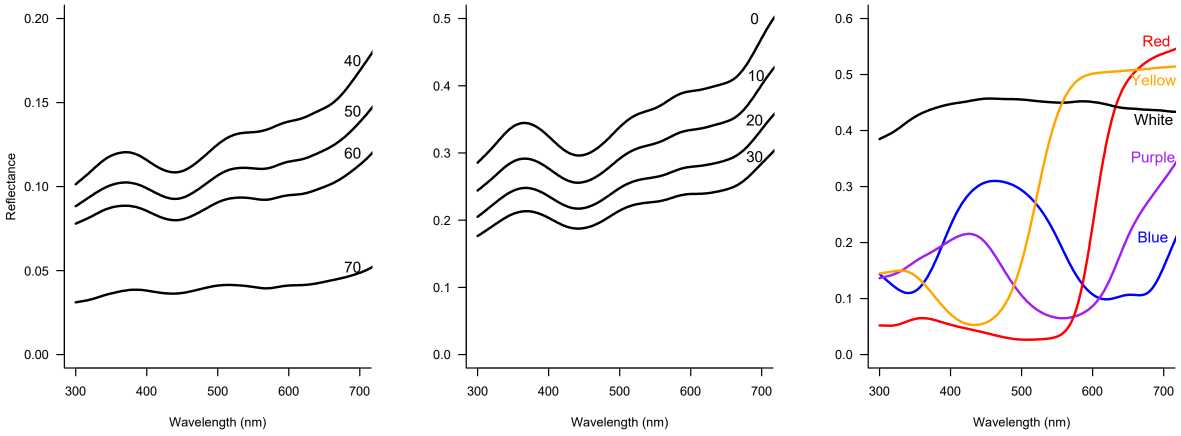

Fig. S3. Reflectance spectra of the different shades of grey and coloured paper. The reflectance spectra were measured with a bifurcated probe under an angle of ~30 degrees to avoid surface reflections. The left and middle panel show the grey shades used in the colour vision experiments using dark and light grey stimuli, respectively; see Fig. 2 of the maint text. The numbers indicate the percentage black ink coverage. The right panel shows the reflectance spectra for the colour trapping in the field, corresponding to Fig. 3 in the main text. The red paper stimulus used in the colour trapping is identical to the red paper that was used in the colour vision experiments.

Dataset 1. Spectral sensitivity numerical data for the two species.

Available for download at
https://journals.biologists.com/jeb/article-lookup/doi/10.1242/jeb.250181#supplementary-data
